# Towards multiple interactions of inner and outer sensations in corporeal awareness

**DOI:** 10.3389/fnhum.2015.00163

**Published:** 2015-04-01

**Authors:** Giuliana Lucci, Mariella Pazzaglia

**Affiliations:** ^1^IRCCS Fondazione Santa LuciaRome, Italy; ^2^Department of Psychology, University of Rome “La Sapienza”Rome, Italy

**Keywords:** body, multisensory information, spinal cord injury, rehabilitation, awareness

## Abstract

Under normal circumstances, different inner- and outer-body sources are integrated to form coherent and accurate mental experiences of the state of the body, leading to the phenomenon of corporeal awareness. How these processes are affected by changes in inner and outer inputs to the body remains unclear. Here, we aim to present empirical evidence in which people with a massive sensory and motor disconnection may continue to experience feelings of general body state awareness without complete control of their inner and outer states. In these clinical populations, the activity of the neural structures subserving inner and outer body processing can be manipulated and tuned by means of body illusions that are usually based on multisensory stimulation. We suggest that a multisensory therapeutic approach could be adopted in the context of therapies for patients suffering from deafferentation and deefferentation. In this way, these individuals could regain a more complete feeling and control of the sensations they experience, which vary widely depending on their neurological condition.

Normally, we have a sense of our own body, and our corporeal awareness is related to the integration of information from both outside and inside the body. Structural alterations and systematic misperceptions of body awareness are due to changes relative to inner and outer experiences during pathological and physiological conditions. In this article, we discuss the conscious body-self phenomenon from a neurological perspective. Our aim is to clarify the degree of body/brain continuity required to support global corporeal awareness in patients with some form of detachment, as seen in locked-in syndrome (LIS) or spinal cord injury (SCI). Finally, we highlight the mutual interplay of multisensory integration across exteroceptive and interoceptive domains with regards to modulating bodily awareness disorders and increasing the coherence of sensory experiences from one’s own body.

## Inner and Outer Sources of the Body State

Intuitively, the interoceptive system is the first source that contributes to body consciousness by receiving signals from the internal milieu and relative to homeostatic status. This inner source may reflect our most “primitive” feelings about the needs of the “soma”, which are structured within composite and dynamic maps that are updated moment to moment depending on changes in the inner body (Craig, 2003; Seth et al., 2012). In particular, at the brain stem level, autoregulatory centers (such as the nucleus of the solitary tract, the periaqueductal gray, the parabrachial nucleus, the postrema area, and the hypothalamus; (Damasio, 1993) send signals related to visceral sensations to the insular cortex and interoceptive centers (Craig, 2009). These inputs mediate the signals of subjective evaluation of the internal bodily state within the closely interconnected orbitofrontal cortices (Devue et al., 2007). The momentary entity of the “*physical self*” (the sense of the physiological condition of the body; (Craig, 2002), “*sentient self*” (the subjective awareness of oneself as a feeling entity; (Craig, 2009), “*neural self*” (the primordial representations of the individual’s body; (Damasio, 1993), and the “*behavioral agent*” (affective motivations of the volitional agent; (Craig, 2002) emerge in these cortices and attend to inner bodily feelings (Critchley et al., 2004; Craig, 2009) and homeostatic states (Craig, 2003; Seth et al., 2012). Along with the vestibular system, the proprioceptive system completes the internal picture of body consciousness via the perception of physical sensations related to position and movement.

**Figure 1:**
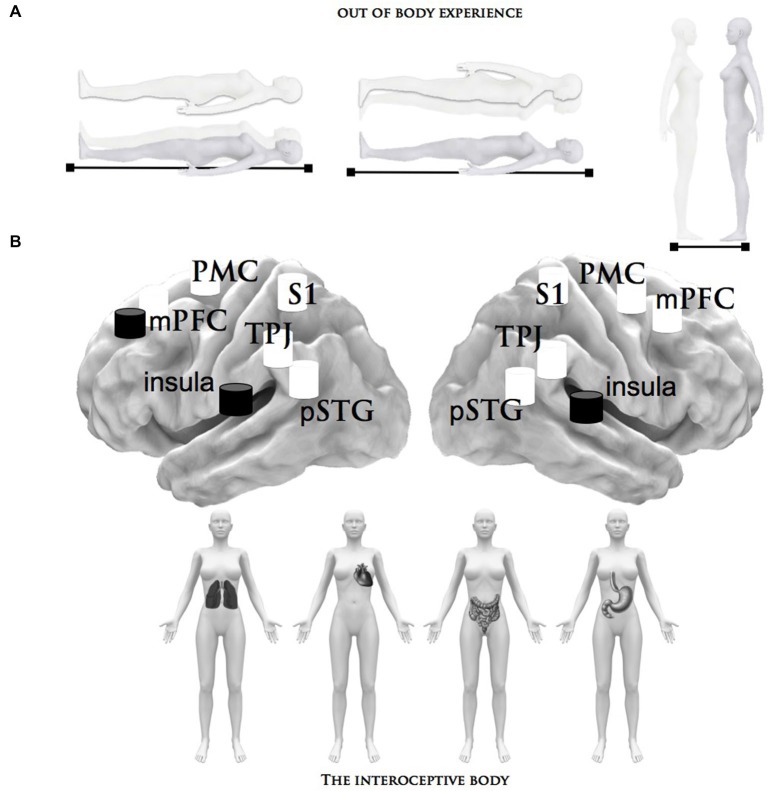
**Summary of the brain structures that process bodily signals**. This figure describes the neural network related to the inner and outer body states that can be manipulated and tuned by different factors: **(A)** Depiction of the phenomenology of out-of-body experiences, in particular the autoscopic experience of seeing a double-self without the experience of leaving one’s own body. **(B)** The interoceptive stimuli that have been shown to activate the neural structures subserving inner body processing: the heartbeat and respiratory perception/detection and the distension of the bladder and stomach. The central brain illustration depicts the critical brain areas related to the inner (black) and outer (white) body signals, which are alluded to in these paradigms; pSTG = posterior superior temporal gyrus, TPJ = right temporoparietal junction, S1 = primary somatosensory cortex, mPMC = medial premotor cortex, insula, mPFC = medial prefrontal cortex.

The outer source, the second source of corporeal awareness, may rely on the flow of co-perceived multisensory information (in this case, visual, somatosensory and, at least in part, proprioceptive signals) that define the physiological condition of the external body (Aglioti and Pazzaglia, 2010, 2011; Blanke, 2012; Serino et al., 2013). Accordingly, body-state awareness should be related to the brain areas that receive sensory information about the body and, ultimately, initiate motor reactions. The most likely candidates are the posterior somatosensory cortex, the left temporoparietal cortex and, to a lesser extent, the occipitotemporal cortex and the motor cortex, which guides somatic motor activity (Blanke, 2012). This outer source is known to predict that major alterations in corporeal awareness depend on changes relative to efferent (motor) and afferent (sensory) experiences. These modifications can be measured during certain experimental conditions (Botvinick and Cohen, 1998; Lenggenhager et al., 2007; Blanke and Metzinger, 2009), as well as in patients with a number of neurological and psychiatric diseases and disorders (Bermudez, 1998; Gallagher, 2005). Neuroscientists have investigated the central mechanisms of corporeal awareness by using body surrogates in virtual reality and robotics technologies (Slater et al., 2010; Maselli and Slater, 2013) and providing subjects with ambiguous multisensory information about body reduplication (Ehrsson, 2007). These studies, inspired by the autoscopic phenomena investigated in clinical populations (Devinsky et al., 1989; Blanke et al., 2004; Bolognini et al., 2011), suggest that the adult human brain is equipped with neural systems in which the point of view for the conscious awareness of the body can be manipulated. This phenomenon is generally referred to as an out-of-body experience (Muldoon and Carrington, 1929; Ehrsson, 2007). During autoscopic and heautoscopy hallucinations, patients see a duplication of their body in an extracorporeal position (see Figure 1). Although the precise origin of out-of-body experiences has not yet been identified, it has been proposed that the feeling of being outside the real body can be due to a disintegration of the inner signals in temporo-parietal-insular brain and a failure to produce coherent, central integration bodily signals (Blanke and Mohr, 2005). Under this abnormal multisensory interplay, the consequence is a complex disturbance of perception and conception of corporeal perspective and physical location.

The illusory alterations of awareness of the body-self can be induced experimentally during the so-called “rubber hand illusion” (RHI), where a dummy hand is used to create a multisensory conflict (Serino et al., 2013). In this paradigm, the synchronous stroking of an observed fake hand and one’s own hidden hand leads to an illusory sensation of touching one’s own hand (Botvinick and Cohen, 1998). This multisensory manipulation induces changes not only in one’s own touch perception, but also in terms of inner perceptions such as skin temperature (Salomon et al., 2013), the heartbeat (Suzuki et al., 2013), and pain sensations (Hansel et al., 2011). Phenomenologically, it is possible to induce the conscious experience of self-identification, self-location and a first-person perspective of inner and outer bodily signals through an enhanced multisensory experience (Blanke, 2012).

## Corporeal State Awareness in an Insentient and Unmoving Physical Body

This perspective article will consider some of the scientific work that has been focused on patients with both massive somatic deafferentation and motor deefferentation of the body in order to investigate pathological perspectives of corporeal state awareness. We chose to focus on disturbed outer and inner body processes in SCI in order to investigate how they are manifest in terms of general awareness of the body. In patients with SCI, sensorimotor traffic between the body and the brain is typically interrupted. This disconnection leads to no sensation in, or voluntary movement of, either the lower body or the entire body. The neurological status, and the level and severity of damage to the spinal cord, determines the number of organs and segments of both the inner and outer body that are “isolated” from the brain and the various degrees of sensory-motor loss (Hohmann, 1966; Montoya and Schandry, 1994). Following SCI, spinal somatic visceral pathways are partially or totally disrupted, and internal perception is distorted (Salvioli et al., 2012). Such alterations to visceral perception include gastrointestinal (Miller and Fenzl, 1981) and intestinal (Salvioli et al., 2012) malfunctions, reduced cardiac awareness (Montoya and Schandry, 1994) and sensations of systematic bladder-filling (Ersoz and Akyuz, 2004). Individuals with an SCI may have difficulty in identifying primitive bodily sensations, e.g., hunger and thirst (Lenggenhager et al., 2012), presumably due to disturbances in somato-visceral afferent feedback (Haas and Geng, 2008). Unfortunately, after injury, many of the inner and outer sensations of the body present as pain. Pain is something that, when felt in an insentient and unmoving body part, promotes one of the most basic sensory experiences of body awareness. As one patient with SCI explained: “The pain, is the connection with the body—the pain is my friend” (Cole, 2004). Here, pain serves as a physical entity that allows for the possible feeling of the entire physical body rather than nothing and numbness (Cole, 2004).

Moreover, sensory loss in parts of the outer body modulates an individual’s corporeal awareness of how those body parts “feel”. When somatic information throughout the body is not complete, individuals, such as patients with SCI, tend to touch themselves regularly to reassure themselves that they do indeed have a body and that they actually exist (Cole, 2004; Lenggenhager et al., 2012). Therefore, in the absence of vision, a complete inability to utilize proprioceptive information impedes proper postural control and spatial perception of body position, which are necessary for immediate sense of self-attribution (Gallagher, 2000; Galli and Pazzaglia, 2015). In addition to the loss of sensation and the sense of joint position, patients with SCI tend to experience a relevant loss of limb movement. Moreover, these patients tend to have a disruption in the intentional control of movement below the injury level. A real sense of acting in/on the world is crucial to expressing physical self-awareness. The absence, and sometimes altered presence, of relevant sensory signals, and a profound alteration of movement have relevant effects on the automaticity of body awareness (Cole, 2004). The state of the body is, however, not completely passive, and new and different relations with one’s own body in terms of movement can be actively learned (Pazzaglia et al., 2013). Additionally, the absence of any perception seems to shift the underlying mechanisms of corporeal awareness to the preserved visual and vestibular systems. While this representation in individuals with SCI is abnormal for the specific body region, it does not disappear for the body as a coherent whole (Fuentes et al., 2013). Even in the most extreme case of brain-body disconnection, such as LIS, the brain maintains a relatively persistent representation of the body (Nizzi et al., 2012). Acute ventral pontine lesions are the most common cause of the quadriplegia that characterizes LIS, with patients exhibiting total large-fiber deafferentation and deefferentation below the neck. These individuals are classically described as being “trapped” within their paralyzed bodies (Bauer et al., 1979; Inci and Ozgen, 2003; Laureys et al., 2005). Obviously, sensations from the body constitute a major element of the experience of awareness. However, despite the fact that global paralysis clearly restricts these patients’ capacity to feel and move their body, their internal representation is not disembodied (Kyselo and Di Paolo, 2013). In cases of LIS, the global corporeal awareness can be linked to minimal eye movements, which are capable of actively engaging the whole body (Nizzi et al., 2012). Visual image of the body can continuously update insentient body segments (Gallagher and Cole, 1995). Therefore, visual experience is dominant (Pazzaglia, 2015) and independent of the loss of sensory and motor inputs/outputs. Consequently, it is not surprising that images can elicit an immediate “reconnect” of body-self awareness. As one patient with an upper cervical injury and a complete loss of all movements and sensations of body said: “I still view my body as whole; it’s just motionless. I’m not a head on a bag of potatoes. I still know it is there. I like to see it. It is still me and I am still it. Totally. I always say at the end of the day that I am motionless but I am still me, body and all” (Cole, 2004). In this patient and others, visual monitoring and visual memory keep body awareness intact. The brain can construct a sense of wholeness and configuration based on memorized body data established during years of life. This type of awareness may reflect an innate configuration within certain brain regions that is organized in such a way that the body is represented as a coherent whole, rather than separately for each body part. Despite the massive sensory and motor loss, the phantom limb and RHI experiences in SCI (Burke and Woodward, 1976; Lenggenhager et al., 2012) and LIS (Tanaka et al., 2008) patients support the idea that the complete body image is maintained despite alterations in neurological function. It is possible that from the adult brain, the idea of a body that is brutally interrupted does not exist, and visual monitoring may be helping patients to recreate a coherent body image.

## Multisensory Interaction Generates a New, More Precise, Definition of the Body in Clinical Populations Suffering from Deafferentation

Together, clinical examples provide some empirical support for the notion that the state of the body is, in fact, a synthesis of multiple and complex states, and that offline and online body representations may be invoked to obtain a coherent and accurate mental experience of corporal awareness. Consequently, a lack of any movement and sensation in these patients probably has more relevance for the control of inner and outer states than it does for general awareness of the body-self. It remains unclear whether, in the absence of new sensory inputs: (i) it is possible to update one’s own body representation (Serino et al., 2013); and (ii) the body, like the brain, is able to adapt (Kyselo, 2014). However, the sensorial and motor loss of a limb and, consequently, of functionality, can result in bodily changes that adjust what is known as the internal body map (Pazzaglia et al., 2013).

Interestingly, in patients with tetraplegia, when the phenomenological experience of the body illusion is induced experimentally during the RHI, it enhances bodily feelings related to deafferented body parts. During multisensory conflict, these patients unexpectedly attribute vivid tactile and proprioceptive sensations to completely numb fingers (Lenggenhager et al., 2013). Delivering tactile stimuli to the non-deafferented fingers can increase the sense of having a complete physical body, and thus hints at the plastic remapping of numb finger representations. In a similar vein, stroking the face of deafferented patients evokes the illusory perception of one’s own numb hand, suggesting a possible remapping of hand-face representations (Tidoni et al., 2014). The “mislocalization” of light touch to the numb hand suggests a strong capture of global body-self awareness. This increased malleability of the sense of one’s body results from both dampened interoceptive perception and afferent multisensory signals that are remapped or augmented during multisensory illusory interplay. Sensation loss can re-emerge during stimulation, and the perceived illusory body may reflect an isomorphic corporal representation to the innate body. It is worth noting that patients with SCI do have residual capacity in some internal and external body parts. After injury, spared axons sprout and make new connections and a small portion of the spinal cord remains intact, even in cases of severe and complete spinal cord lesions(Anderson et al., 2004; Guest et al., 2005). Thus, small sensory fibers could provide reduced sensations in insentient body regions (Cariga et al., 2002).

Although the sensations are altered and often discontinuous, the operations of the actual body are nevertheless mapped in the body’s neural structures (Fuentes et al., 2013; Pazzaglia et al., 2013). The mismatch between the body viewed and not felt, and the proprioceptive and interoceptive information of limited body parts, nonetheless provides a partial signal to the relevant brain area (Lenggenhager et al., 2013; Tidoni et al., 2014). This allows a feeling of awareness of the body-self despite the radical bodily change. During multisensory stimulation, enhanced signals in the neural “body-sensing” region of what one’s body felt like prior to injury re-emerge in terms of what one’s actual body feels like following injury (Lenggenhager et al., 2013; Pazzaglia et al., 2013). This recreates reliable and coherent bodily awareness. Multisensory integration provides a significant opportunity to promote neural plasticity, which could optimize feelings that are linked to corporeal awareness. Patients with chronic conditions like tetraplegia or LIS may ultimately profit from multisensory therapy augmented by artificial bodies. Accordingly, it is possible to induce dynamic and capable manipulations of body awareness by extending perceptual sensations from sentient regions to numb body parts using surrogate body or salient extracorporeal devices that patients can move and control as if they were their own (Pazzaglia, 2015). This could potentially provide patients with not only the ability to experience self-enhanced sensations, but to also be able to once again feel inner, insentient, and extending body parts as their own. The confluence of the multimodal approach and new technologies (e.g., body interfaces) used to replace absent movements and sensations could, in the near future, improve the embodiment of the body regions that patients are unaware of.

## Anticipatory Coding of Body Awareness Based on the Interaction of Inner and Outer Signals

If bodily self-consciousness depends on how the brain dynamically processes multisensory bodily signals (Blanke, 2012), then different body states may also be induced by manipulating internal signal processing (Seth, 2013). These hypotheses find strong support in, for example, cardio-visual experiments in which heartbeat-visual synchronization, which is similar to the more well-known visuo-tactile synchronization performed during the RHI, modulates bodily self-consciousness and tactile perception (Tsakiris et al., 2011; Aspell et al., 2013; Suzuki et al., 2013).

Moreover, visuo-respiratory stimulations modulate the feeling that the motor act of breathing is associated with the virtual body. During synchronous stimulation in the RHI, subjects perceive a change in breathing location; in other words, they “locate their breathing” at a point closest to the virtual body (Adler et al., 2014). These data suggest that interoceptive awareness seems to be modulated in multisensory contexts.

Yet, visual capture not only modulates interoceptive awareness under conditions of multisensory integration, but also has an impact on shared representations in the motor domain (Pazzaglia, 2013). For example, compared to poor heartbeat perceivers, good perceivers are *more* inclined to the automatic imitation of the movements of others (Ainley et al., 2014).

In view of this, a fundamental, but far less addressed, issue concerns the predictive control of interoception, in which the brain tries to minimize the discrepancy between states of subjective feeling and the effect predicted for the interoceptive state of the body (Seth et al., 2012; Seth, 2013). For example, if a subject’s sensitivity to breathing is altered, the presentation of a periodic external stimulus (e.g., tones or lights) can lead to them synchronizing their own breathing with the presented stimulus. This technique would allow patients to compensate for distorted perceptions by capturing real respiratory cues, thereby allowing for the correct awareness of the breathing activity. More specifically, a subject would be required to use an anticipatory code of the perceived outcome to intentionally select their breathing rate rather than merely feeling it. This predictive coding allows patients to congruently adapt their inner bodily signals to their outer ones. In other words, interoception sensitivity is controlled by anticipatory effects.

Predictions about expected and unexpected future interceptive perceptions inform the body of corresponding inner malfunctions. The analysis of how inner and outer sources contribute to the perspective coding of the body in clinical patients remains a fundamental topic for future research.

## Concluding Remarks

According to the laws of multimodal contingencies and the active engagement of the conscious feedback generated by the interoceptive state, the cortical systems devoted to bodily self-consciousness seem to be susceptible to adjustment.

**Figure 2:**
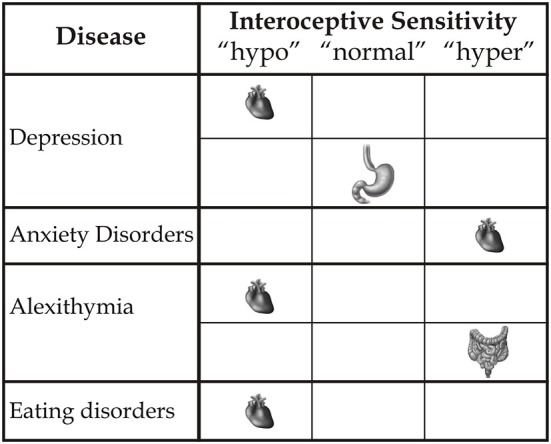
**This figure represents how different clinical disorders influence inner bodily self-consciousness in terms of hypo-, normal or hyper-interoceptive sensitivity, specifically related to the heart, stomach and intestines**.

Therefore, multimodal augmented training can reveal benefits not only in terms of outer signals, such as motor functions, but also with respect to inner signals, as documented in experimental conditions (Tsakiris et al., 2011; Aspell et al., 2013; Suzuki et al., 2013; Adler et al., 2014), and clinical conditions in relation to, for example, the reduction of neuropathic pain (Villiger et al., 2013a,b). Although neuroscience studies referring to the processing of the body’s outer signals seem to predominate, the inner body is a central aspect of awareness of the body-self. However, what is needed is a more precise definition of the different interoceptive variables that influence the modulation of the body state and induce changes based on a multisensory approach. This distinction is important with respect to considerations related to etiology, research and, crucially, the development of new rehabilitative treatment models with respect to different clinical deficits and diseases. Alterations of interoceptive awareness are present in many psychological clinical states (see Figure 2). For example, anxiety is associated with a high interoceptive accuracy level (Domschke et al., 2010), while depression (Mussgay et al., 1999; Dunn et al., 2007), eating disorders, (Pollatos et al., 2008; Paulus and Stein, 2010; Klabunde et al., 2013), alexithymia (Kano et al., 2007; Herbert et al., 2011), and aging (Khalsa et al., 2009) are related to a diminished interoceptive ability. However, new therapeutic clinical strategies linked to interoceptive states remain a challenge. We argue that further focus on the plasticity of the neural structures assigned to inner body processing can help to formulate a novel concept of body-state awareness and add to both fundamental processes and the multisensory side of rehabilitative treatments.

## Conflict of Interest Statement

The authors declare that the research was conducted in the absence of any commercial or financial relationships that could be construed as a potential conflict of interest.
